# MXene Enhanced 3D Needled Waste Denim Felt for High-Performance Flexible Supercapacitors

**DOI:** 10.1007/s40820-023-01226-y

**Published:** 2023-11-29

**Authors:** Wei Fan, Qi Wang, Kai Rong, Yang Shi, Wanxi Peng, Handong Li, Zhanhu Guo, Ben Bin Xu, Hua Hou, Hassan Algadi, Shengbo Ge

**Affiliations:** 1 Key Laboratory of Functional Textile Material and Product of the Ministry of Education, School of Textile Science and Engineering, Institute of Flexible electronics and Intelligent Textile, Xi’an Polytechnic University, Xi’an, 710048 People’s Republic of China; 2 Co-Innovation Center of Efficient Processing and Utilization of Forest Resources, College of Materials Science and Engineering, Nanjing Forestry University, Nanjing, 210037 People’s Republic of China; 3 Henan Province International Collaboration Lab of Forest Resources Utilization, School of Forestry, Henan Agricultural University, Zhengzhou, 450002 People’s Republic of China; 4 Integrated Composites Lab, Department of Mechanical and Construction Engineering, Northumbria University, Newcastle Upon Tyne, NE1 8ST UK; 5 College of Materials Science and Engineering, Taiyuan University of Science and Technology, Taiyuan, 030024 People’s Republic of China; 6 Department of Electrical Engineering, Faculty of Engineering, Najran University, 11001 Najran, Saudi Arabia

**Keywords:** MXene, 3D needled waste denim felt, Supercapacitors, Carbonization

## Abstract

MXene, a transition metal carbide/nitride, has been prominent as an ideal electrochemical active material for supercapacitors. However, the low MXene load limits its practical applications. As environmental concerns and sustainable development become more widely recognized, it is necessary to explore a greener and cleaner technology to recycle textile by-products such as cotton. The present study proposes an effective 3D fabrication method that uses MXene to fabricate waste denim felt into ultralight and flexible supercapacitors through needling and carbonization. The 3D structure provided more sites for loading MXene onto Z-directional fiber bundles, resulting in more efficient ion exchange between the electrolyte and electrodes. Furthermore, the carbonization process removed the specific adverse groups in MXenes, further improving the specific capacitance, energy density, power density and electrical conductivity of supercapacitors. The electrodes achieve a maximum specific capacitance of 1748.5 mF cm^−2^ and demonstrate remarkable cycling stability maintaining more than 94% after 15,000 galvanostatic charge/discharge cycles. Besides, the obtained supercapacitors present a maximum specific capacitance of 577.5 mF cm^−2^, energy density of 80.2 μWh cm^−2^ and power density of 3 mW cm^−2^, respectively. The resulting supercapacitors can be used to develop smart wearable power devices such as smartwatches, laying the foundation for a novel strategy of utilizing waste cotton in a high-quality manner.

**Supplementary Information:**

The online version contains supplementary material available at 10.1007/s40820-023-01226-y.

## Highlights

- An ultralight and flexible supercapacitor is developed by an effective 3D fabrication method that uses MXene to fabricate waste denim felt through needling and carbonization.
- The electrodes have a maximum specific capacitance of 1748.5 mF cm^−2^ and demonstrate remarkable cycling stability with more than 94% after 15,000 galvanostatic charge/discharge cycles
- The loaded more MXene onto *Z*-directional fiber bundles results in enhanced specific capacitance, energy density and power density of supercapacitors.

**Supplementary Information:**

The online version contains supplementary material available at 10.1007/s40820-023-01226-y.

## Graphical abstract

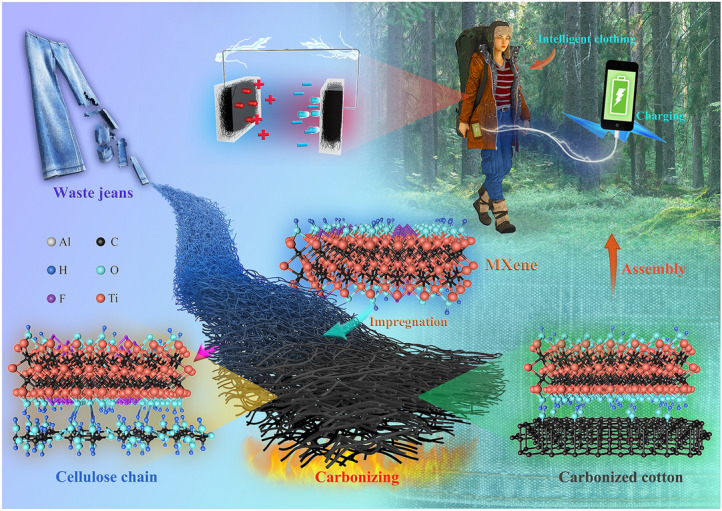

## Introduction

Two-dimensional (2D) materials, including disulfides (MoS_2_, ZnS), graphene and transition metal carbides/nitrides (MXene), possess stratified structures that have promising potential for energy storage [1–4]. The MXene possesses tailorable surface chemistry [5], electrical conductivity (approximately 15,000 S cm^−1^) [6], high volumetric capacitance (approximately 1500 F cm^−3^) [7], excellent mechanical properties [8] and large interlayer spacing. Given these properties, a high-performance supercapacitor can be made with MXene as an electrode material [9, 10].

The first transition metal carbides/nitrides (M_n+1_X_n_, N = C or N, M = V, Ti, Nb, Cr, Zr, Ta or Hf) were reported in 2011 by Naguib et al. [11] using Ti_3_C_2_ and Ta_4_C_3_ wet etching method. Given the emergence of smart wearable technology, this work opened up the possibility of forming a series of M-atoms 2D structure materials. At present, the question of how MXene can be combined with flexible substrates (i.e., fiber, film or fabric) to meet the needs of supercapacitors or other energy storage devices merits further research [12, 13]. There have been attempts to manufacture MXene/cotton fabric [14], MXene/cotton yarn [4], MXene/carbon cloth [15] and MXene/silver-plated nylon fiber [16]. However, the small area of these 2D substrates may result in a decreased electrochemical performance in the electrodes of supercapacitors due to low MXene loading. As a result, electrodes must be loaded with high levels of MXene to achieve the desired electrochemical performance. The use of 3D flexible substrates might be an appropriate choice since they ensure sufficient contact between electrodes and electrolytes during the charge/discharge process in supercapacitors [17, 18]. In addition, a 3D flexible structure substrate could significantly extend the life of devices by preventing the loss of active materials inside the electrodes [19].

Among flexible substrates, textiles are often used as raw materials. Unfortunately, the production of textile products burdens the environment by occupying land, consuming water, using pesticides and emitting greenhouse gases [20, 21]. As a result of rapid growth in the textile industry and production, unwanted denim fabrics has become one of the most common textile wastes that create environmental pollution and problematic disposal [22]. Therefore, combining textile wastes with MXene to create flexible supercapacitors with excellent electrical conductivity is hypothesized to address the disposal problem [23]. Nevertheless, the supercapacitors with textile-derived substrates should consider their low electrical conductivity since this will negatively influence their electrochemical performance during operation [24].

Supercapacitors have attracted much attention as a typical energy storage material due to their fast charging–discharging process, high power density, long cycle life and wide range of working temperatures, which present excellent application prospects. However, the lower actual capacitance and energy density in the reported works limit their practical application to a great extent. Thus, effective means to enhance the electrochemical performance of supercapacitors are considered a research hot spot.

In light of the above issues, this work demonstrates the preparation of high-performance flexible electrodes and supercapacitors from MXene (the active material) and 3D needled waste denim felt (the flexible substrate) via impregnation and carbonization. As a result of the carbonization process, it is anticipated that denim felt would have an improved electrical conductivity. This would further enhance the efficiency of the supercapacitors by allowing ions/electrons to be transported more efficiently during the charging process. Additionally, several specific groups that negatively affect the electrochemical behavior of MXene could be removed at high carbonization temperatures, thereby increasing the energy storage capacity for supercapacitors. Therefore, the proposed strategy would reveal a potential transformation of textile waste into a promising supercapacitor for application in smart wearable devices.

## Experimental Section

### Raw Materials

Waste denim felt was offered by Guangzhou Xinyang Textile Co., Ltd., whereas Ti_3_AlC_2_ (the precursor of MXene) was supplied by 11 Technologies Co., Ltd. The lithium fluoride (LiF), polyvinyl alcohol (PVA), phosphoric acid (H_3_PO_4_) and hydrochloric acid (HCl) were obtained from Shanghai Aladdin Biochemical Technology Co., Ltd., Xi’an Ruilijie Experimental Instrument Co., Ltd., Tianjin Kemiou Chemical Reagent Co., Ltd. and Sinopharm Group Chemical Reagent Co., Ltd., respectively.

### Preparation Route of MXene

**Fig. 1:**
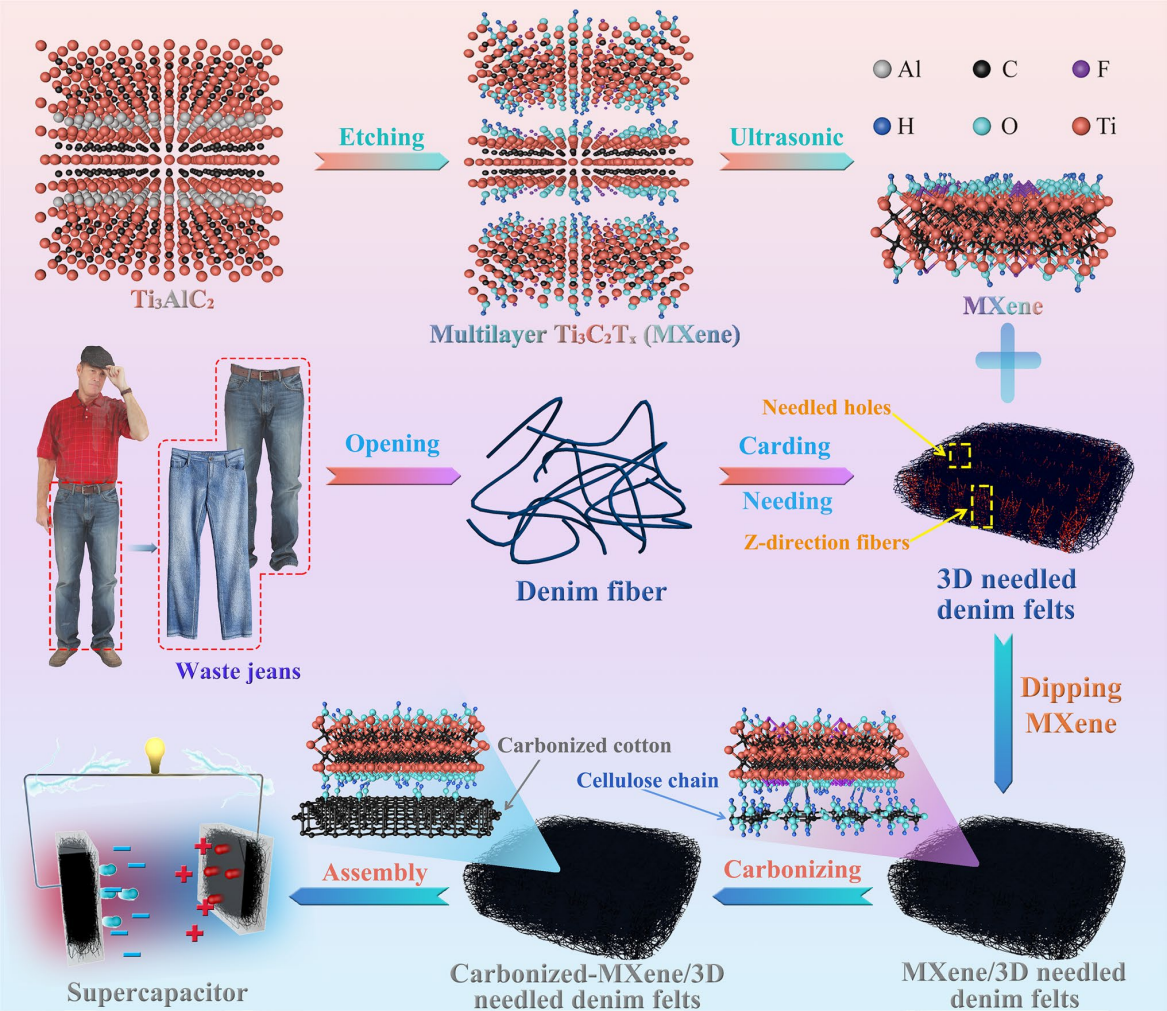
Preparation route of the MXene/3D needled denim felts as electrodes and supercapacitors

The preparation of MXene was initiated by etching the aluminum layer of Ti_3_AlC_2_ with a mixed solution of LiF and HCl. Firstly, 10 mL HCl (9 M) was added to 0.8 g LiF for a 10-min chemical reaction to prepare the etchant. Then, Ti_3_AlC_2_ was added into the etchant solution slowly to obtain the acidic suspension. Afterward, the suspension was magnetically stirred for 48 h at 40 °C and then rinsed with deionized water multiple attempts until almost neutral (pH ~ 7). Finally, the obtained suspension was subjected to an ice bath for 6 h ultrasonic vibration followed by 30-min centrifugation in an Ar atmosphere to yield a stable supernatant of dark green MXene. Figure 1 displays the preparation route of MXene until its application as a supercapacitor.

### Preparation of Carbonized MXene/3D Needled Denim Felts (CMDF)

3D needled waste denim felts (DF) was sourced from waste jeans via the cutting–opening–carding–needling process (Fig. 1), and more details about the preparation method can be found elsewhere [25]. The DF with an area density of 0.022 g cm^−2^ was chosen as the substrate. After needling, DF samples were cleaned with acetone and deionized water to remove pollutants.

Various MXene suspension concentrations (2%, 4%, 6% and 8%) were adopted to obtain MXene/3D needled waste denim felts (MDF) with high MXene load. The loadings of MXene are shown in Fig. S1. Initially, the DF samples were immersed thoroughly in the prepared MXene suspension for 10 min. Then, DF samples were fished out and dried at room temperature for 120 min. The dried MDF was carbonized in a tube furnace at different temperatures (800, 1,000 and 1,200 °C) under a heating rate of 5 °C min^−1^ for 60 min in an Ar environment to acquire CMDF. On the other hand, the DF (without MXene coating) was directly carbonized in a tube furnace to yield carbonized 3D needled denim felts (CDF) for comparison. The initial thickness of the DF was measured at 1.5 mm, which was subsequently carbonized to produce CMDF with a thickness of 0.7 mm. The MXene morphologies present on both the initial DF and the resulting CMDF are shown in Fig. S2.

### Characterizations

The microstructure and surface morphology were inspected by transmission electron microscopy (TEM, JEM-2100F, Japan) and scanning electron microscopy (SEM, Quanta 450FEG, USA). The X-ray diffraction technology (XRD, D8 Advance, German) was utilized to investigate the sample phase compositions, while a inVia Reflex micro-Raman spectroscopy system was adopted to produce Raman spectra. X-ray photoelectron spectroscopy was utilized to examine the sample chemical components. The thermal decomposition process of samples (from 50 to 1200 °C) was evaluated by a thermal analysis infrared mass spectrometer under an N_2_ environment at a thermal rate of 10 °C min^−1^. The water contact angle of samples was investigated and calculated via the sessile drop method and SCA 20 software. Figures S3-S5 show the SEM images, electrical conductivity, fiber diameters and MXene load in MDF under different concentrations of MXene solution. It was determined that the optimal MXene solution concentration in this work was 6%.

### Measurement of Electrochemical Properties

The electrochemical impedance spectra (EIS), cyclic voltammetry (CV) curves and galvanostatic charge/discharge (GCD) curves of CMDF supercapacitor and electrode devices were measured by the electrochemical workstation (CHI660E, China), and the cyclic GCD curves were conducted on the battery test system (CT2001A, China). Thereinto, a three-electrode system was chosen to measure the electrochemical performance of CMDF electrodes, where the working electrode was CMDF, one molar of H_2_SO_4_ served as an electrolyte, the counter electrode used was Pt and the reference electrode was Ag/AgCl.

As for supercapacitor devices, a double-electrode system with symmetrical CMDF electrodes was applied. Two pieces of CMDF were evenly covered by the PVA- H_3_PO_4_ gel electrolyte and dried under an ambient environment, then overlapping and wrapping PDMS outside as a covering layer.

The specific capacitance area (*C*_*e*_, F cm^−2^) of the CMDF electrode is computed based on GCD curves [26]:

1 $$ C = 2i \times \Delta t/A \times U $$

where *i* (*A* cm^−2^) represents the current density of GCD tests, *U* (V) represents the voltage window, *A* (cm^2^) represents the electrode working area and *∆t* (s) represents the discharge time.

As for CMDF symmetric supercapacitors, the specific capacitance (*C*_sc_, mF cm^−2^), power density (*P*_sc_, W cm^−2^) and energy density (*E*_sc_, Wh cm^−2^) are computed by the following formula [27]:

2 $${C}_{\mathrm{sc}}=i\times \Delta t/A\times U$$

3 $${E}_{\mathrm{sc}}=0.5\times {C}_{\mathrm{sc}}\times {U}^{2}$$

4 $${P}_{\mathrm{sc}}={E}_{\mathrm{sc}}/\Delta t$$

## Results and Discussion

### Chemical Analysis and Structural Characteristics of MXene and MDF

**Fig. 2:**
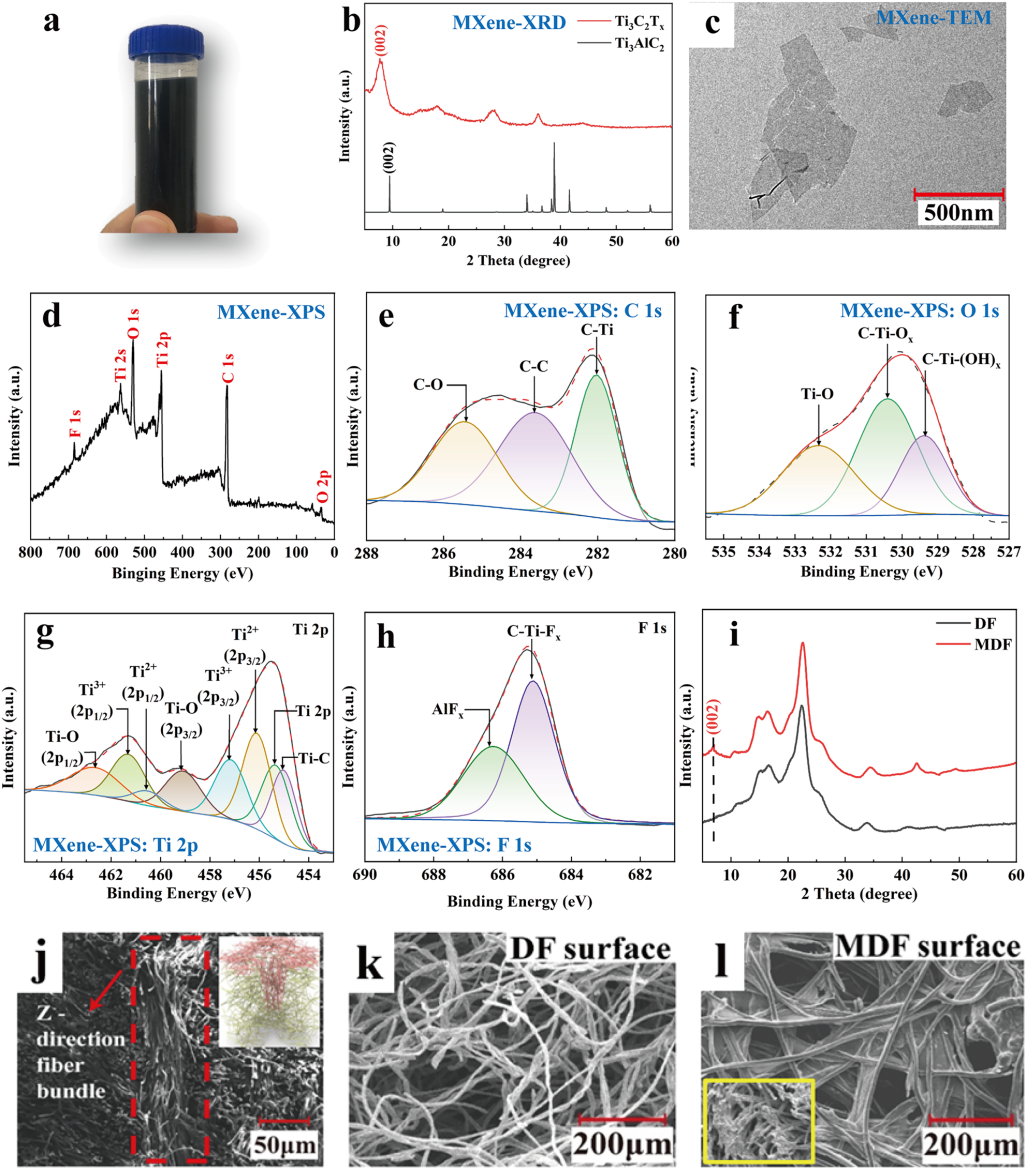
Material characterization results of MXene and 3D needled felts. **a** Optical photograph, **b** XRD pattern, **c** TEM image and **d-h** XPS spectra of prepared MXene. **i** XRD patterns, **j-l** SEM images of the 3D needled waste denim felts and MXene/3D needled waste denim felts

Figure 2 displays the characterization findings on MXene, DF and MDF. The products obtained display supernatant liquid form, suggesting their high stability for subsequent impregnation procedures (Fig. 2a). The XRD peak detected at 7.68° could be ascribed to the typical (002) plane of MXene (Fig. 2b), inferring the successful synthesis of MXene. The TEM image reveals the typical two-dimensional layered structure of the MXene sheet (Fig. 2c), similar to graphene and molybdenum disulfides [28]. Typically, MXene can be synthesized by wet etching of more than 30 different MAX phase precursors, where M is a transition metal element, A is a main group element and X is carbon or nitrogen. MXene obtained in this work displays a nanostructure formed by stripping the Al layer in the MAX phase and attaching –F, –OH and -O to the Ti surface [29]. Spectral analysis reveals the typical peaks for F, Ti, C and O, which concur with the reported chemical components of MXene (Fig. 2d–h). A peak corresponding to a Ti–C bond (282 eV) could be attributed to the core [TiC6] octahedral building block of MXene [30]. Three independent peaks in the O 1* s* spectrum of MXene correspond to the Ti–O bond, C–Ti–O_*x*_ bond and C–Ti–(OH)_*x*_ bond. Several symmetric peaks of the Ti 2*p* spectrum point to the existence of a Ti–C peak around 455.6 eV, which agrees with the XPS measurements. According to the O 1*s* spectrum, the peaks around 459 eV (Ti 2*p*_3/2_) and 462.5 eV (Ti 2*p*_1/2_) are most likely caused by O surface groups with partial surface oxidation (Fig. 2e). The presence of two narrow peaks for F 1*s* spectrum (Fig. 2h) also consistent with the reported works [31]. Therefore, the results above indicate the successful synthesis of MXene.

It has been determined that the MXene has been successfully introduced into MDF based on the presence of a typical (002) diffraction peak located around 7.68°, whereas the peak is not present in DF (Fig. 2i). Figure 2j shows the cross-sectional structure and schematic diagram (the insert image) of DF, revealing the presence of *Z*-direction channels in the thickness direction of DF. Additionally, Fig. 2k highlights the needle hole present on the surface of DF. The *Z*-directional fiber bundles along the thickness direction of the DF would allow MXene to penetrate and fill up DF voids, thereby ensuring complete contact between electrolytes and electrodes. There are obvious attachments of MXene on MDF shown in Fig. 2l and its local enlarged image (the insert image), indicating that MXene is effectively loaded after impregnation.

### Carbonization Mechanism of MXene and Architectural Feature of CMDF

**Fig. 3:**
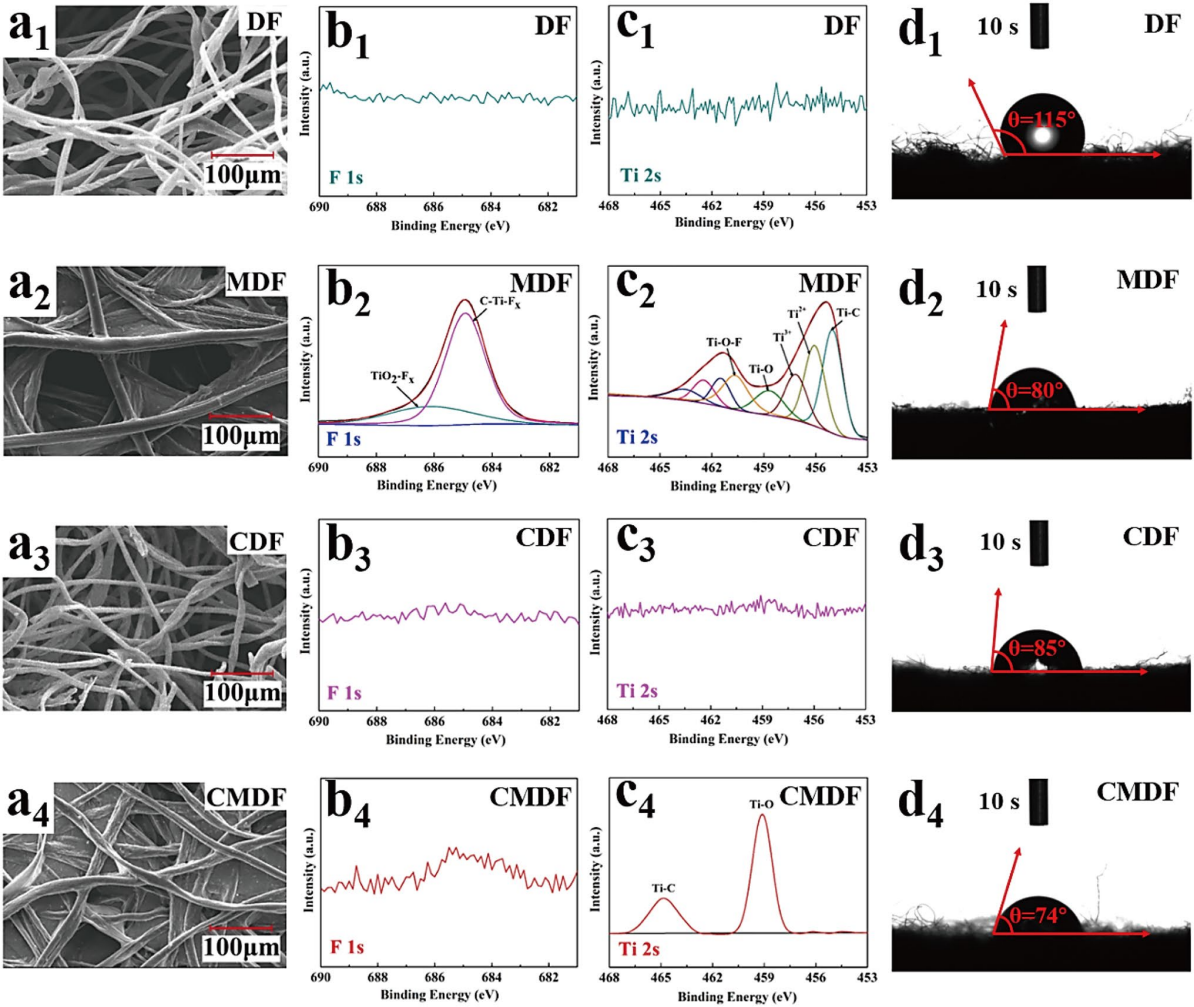
Comparison of SEM image, XPS spectra of F 1s and Ti 2s, water contact angle for different samples. **a1, b1, c1, d1** DF, **a2, b2, c2, d2** MDF, **a3, b3, c3, d3** CDF and **a4, b4, c4, d4** CMDF

The diameter of the fibers in MDF and CMDF that adhere to one another is wider than those of DF and CDF. This is likely due to the effectiveness of the MXene loading during the impregnation process (Fig. 3). There was a negligible effect of carbonization on the needled felt pore structure (Fig. S6). Furthermore, the Ti 2*s* spectrum of MDF is similar to that of MXene while no peak was detected for DF and CDF (Fig. 2d). It is observed that the CMDF presents two typical peaks at 459 eV (Ti–O) and 465 eV (Ti-C), suggesting Ti element was transformed into TiO_2_ and further converted into TiC during the high carbonization temperature. No signal of the F 1*s* spectrum was detected for CMDF, implying the removal of -F groups in MXene during the carbonization process. Figure 3d1–d4 demonstrates the water droplets drop vertically on the surface of samples for 10 s. It was found that the coating of MXene had improved the hydrophilicity of the denim needled felt where the contact angle was reduced from 115° to 80°. At elevated temperatures, the hydrophilic –OH group of cellulose and MXene disappears, yet the carbonized denim fiber attains a fine texture that enhances water transfer [32]. This has led to the reduction in CMDF contact angle due to competitive effects between the two. The consequent increase in hydrophilicity is advantageous for electrolyte penetration, thereby elevating the electrochemical properties of CMDF. Figures S7 and S8 show the EDX mapping and electrochemical test of CMDF under various carbonization temperatures (800, 1000 and 1200 °C).

### Performance of CMDF Electrode

In order to ascertain the most effective MXene loading concentration, various MXene concentration solutions were utilized for verification. As evidenced in Fig. S9, when subjected to the same carbonization temperature, the specific capacitance of 1000CM_6%_DF exceeded that of 1000CM_8%_DF, which in turn surpassed that of 1000CM_4%_DF, with 1000CM_2%_DF being the least effective. In addition, when the current density is increased to 6 mA cm^−2^, the capacitance of the CMDF electrode remains at 888 mF cm^−2^, showing excellent rate performance. The findings obtained can potentially be attributed to the reduction in spacing between neighboring fibers and MXene sheets due to the coated MXene layer. This increases the overall bulk density of the electrochemically active material, thereby affecting its performance. However, it was observed that excessive MXene loading can lead to surface sealing of the DF, thereby hindering the complete contact of the material with the electrolyte, ultimately leading to compromised electrochemical performance. Therefore, it was concluded that a 6% MXene load is optimal under these circumstances.

**Fig. 4:**
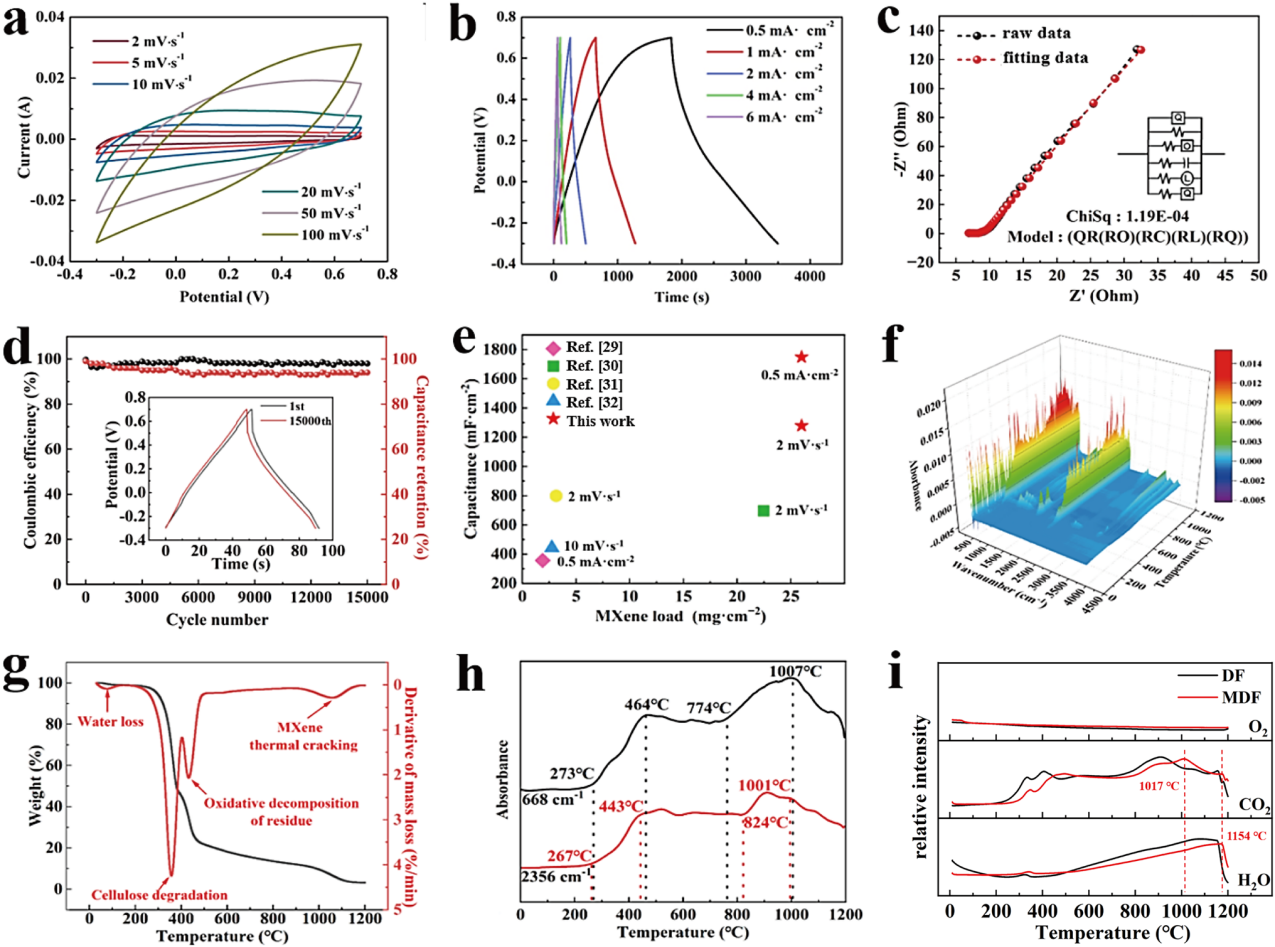
**a** CV graphs under different scanning rates. **b** GCD graphs under different current densities. **c** EIS spectra. **d** Cycling stability (at 6 mA cm^−2^) of the CMDF. **e** MXene load and capacitance of the CMDF with other reported MXene relevant textiles. **f** TG-IR-MS 3D results, **g** TG curves and **h** FTIR spectra of MDF heated from 0 to 1,200 °C. **i** Decomposition gas analysis of DF and MDF during 0 to 1,200 °C

During low scanning rates, CV curves of the CMDF electrode exhibit rectangular symmetric behavior, but at high scanning rates with significant capacitance, they become fusiform due to polarization (Fig. 4a). However, the analysis reveals that CDF exhibits negligible electrochemical properties (refer to Fig. S10), indicating that the electrochemical properties of CMDF are highly comparable to carbonized MXene. Notably, carbonized MXene exhibits a notably larger interlayer spacing than MXene. This is primarily attributed to the generation of TiC and TiO_2_ at high temperatures, which facilitates the entry of the electrolyte into the interlayer, thereby enhancing the electrochemical performance of the material. The curves exhibit a triangle-like structure when the current density increases, indicating good reversibility, and also there are no obvious voltage drops, which further implies high electrical conductivity (Fig. 4b). The optimum specific capacitance of the CMDF (1748.5 mF cm^−2^) was detected at a current density of 0.5 mA cm^−2^, suggesting that CMDF could demonstrate higher performance compared to other MXene supercapacitors. The fitting curves of CMDF show a higher slope at the low-frequency zone than the pristine Nyquist curve, indicating rapid ion diffusion occurs (Fig. 4c). Besides, the CMDF showed a 94% capacitance retention and a 99% coulombic efficiency after 15,000 GCD cycles (Fig. 4d), suggesting that the CMDF has excellent potential as an electrode for high-performance supercapacitors in the future.

This study shows that the MXene load of CMDF is higher than most existing studies, thus demonstrating the structural superiority of 3D needled felt (Table S1, Fig. 4). At 0.5 mA cm^−2^ current density, the CMDF specific capacitance detected was 1748.5 mF cm^−2^ (Fig. 4e) which is significantly higher than the MXene-coated cotton fabric (362 mF cm^−2^) [33]. Furthermore, the specific capacitance of CMDF calculated from CV curves (1290 mF cm^−2^) is also higher than MXene/silk fabric (707 mF cm^−2^) at 2 mV s^−1^ [34], MXene/cotton fabric (794.2 mF cm^−2^) [35] and MXene/carbon fabric (416 mF cm^−2^) at 10 mV s^−1^ [26]. Again, the CMDF electrode with a remarkable specific capacitance offers great promise as a high-performance electrode.

The TG-IR-MS combined system was applied to acquire data of CMDF in real time during 0–1200 °C (Fig. 4f). During the first stage at 83 °C, obvious mass loss is caused by water evaporation while cellulose cracks lead to an increase of the H_2_O, –OH and –O signals at 225 °C (Fig. 4g). The second stage occurs when surface groups of dyes or MXene sheets dissociate slowly at 383 °C into O_2_ and H_2_, with H_2_ being released when the –OH terminals or molecular hydrogen trapped between MXene sheets combine. When the temperature reaches 1017 °C and above (the final stage), MXene begins to crack thermally and gradually releases carbon dioxide by eliminating –F groups. Figure 4h shows the corresponding FTIR spectra of the CMDF. This analysis indicates that the TiO_2_ generation in CMDF begins gradually from 273 °C until it reaches equilibrium between 464 and 774 °C, which corresponds to the release of CO_2_ at 2356 cm^−1^ at 267 °C. Moreover, the F and -OH groups in MXene are continuously removed to generate CO_2_. For the gas productions of DF and MDF during carbonization, the amount of CO_2_ and H_2_O shows significant differences despite their O_2_ spectra almost overlapping (Fig. 4i), which could be ascribed to the existence of MXene.

### Supercapacitor Performance of CMDF

**Fig. 5:**
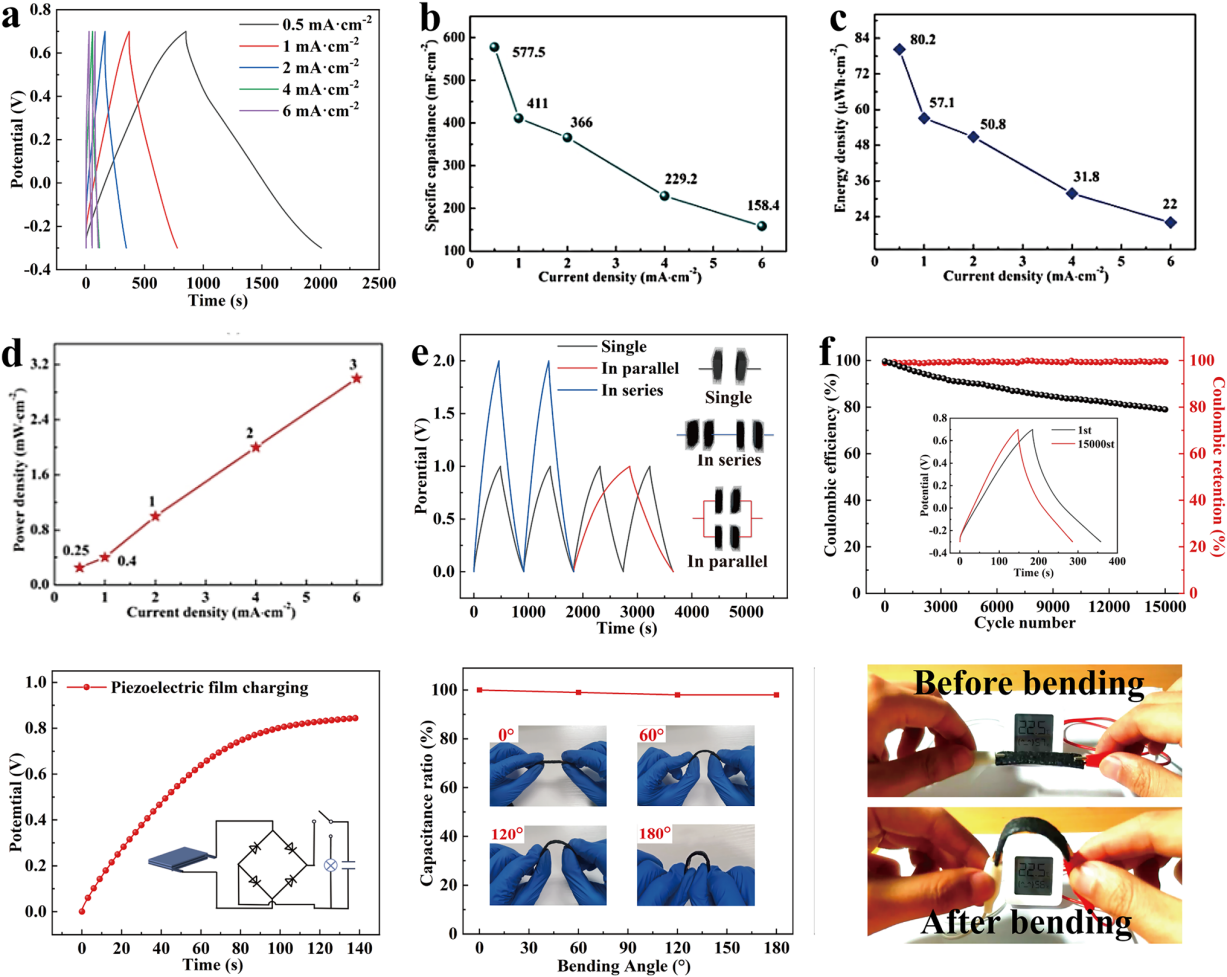
**a** GCD graphs, **b** Specific capacitance, **c** energy density and **d** power density of the CMDF supercapacitor under various current densities. **e** GCD graphs of supercapacitors in series and parallel. **f** Cycling stability of the supercapacitor; **g** charging curve of the supercapacitor to a PVDF film. **h** Capacitance retention of the supercapacitor at various bending angles and **i** the display of the device's application in practice

For the purpose of examining the practical application of CMDF as a flexible energy storage device, two pieces of CMDF were assembled into a symmetric supercapacitor, and the results of electrochemical measurements are shown in Fig. 5. Based on the GCD curves and the symmetrical structure, the supercapacitors show good reversibility, and increased current density reduces the charge–discharge time. On the basis of the GCD curves, the specific capacitance of supercapacitors can be computed (Fig. 5b). Whenever the current density escalates from 0.5 to 6 mA cm^−2^, specific capacitance falls from 577.5 to 158.4 mF cm^−2^. The volume and mass specific capacitance of CMDF are 8.3 F cm^−3^ and 12.3 F g^−1^, respectively, when the current density escalates to 0.5 mA cm^−2^. In addition, the energy and power densities of supercapacitors can also be estimated based on GCD curves. As shown in Fig. 5c, d, energy density gradually decreases from 80.2 to 22 μWh cm^−2^ with escalating current density, achieving the maximum current density of 0.5 mA cm^−2^. Also, there is a reverse trend in power density, which increases from 0.25 to 3 mW cm^−2^.

Compared to a single supercapacitor, two connected devices generate twice the current and specific capacitance (Fig. 5e). Furthermore, two series-connected supercapacitors have a voltage window of 2 V, or twice that of a single supercapacitor, leading to a higher voltage output for the same operating period. This would broaden their operating voltage window when applied to practical applications. Besides, Fig. 5f displays the GCD curves for 15,000 cycles of the supercapacitor at 2 mA cm^−2^, which could evaluate the energy storage durability of the obtained flexible supercapacitor. It can be observed from the results that capacitance retention and coulombic efficiency were maintained after 15,000 cycles at 78.94% and 98%, respectively, indicating remarkable cycling stability.

Flexible supercapacitors can improve energy utilization and provide ultra-long endurance in self-generating systems by collecting electrical energy from electric devices. For this purpose, the SC was connected to a piezoelectric generator. Figure 5g shows that a signal is generated by slapping the film and transmitted to the supercapacitor across a rectifier circuit, which leads to a voltage of 0.84 V after 140 s. Figure 5h shows the capacitance retention of the supercapacitor at various bending angles. Approximately 96% of capacitance retention is maintained whenever the bending angle escalates from 0 to 180 degrees, which indicates excellent flexibility and electrochemical stability. While bending at 180°, the two supercapacitors (1 cm × 2 cm) can still illuminate the electronic screen of the hygrothermograph (Fig. 5i), proving their practical application in smart wearable technology. The experimental results demonstrate that the supercapacitor exhibits remarkable durability against bending and folding, as evidenced by its capacitance retention of 97% and 89.6% (Figs. S11 and S12), respectively, after 1000 cycles of testing.

## Conclusions

This study proposes high-performance electrodes and supercapacitors by combining MXene with 3D needled waste denim felts. It is, therefore, possible to ensure that a high MXene load can be achieved on needled felt by impregnation, while carbonization can remove adverse functional groups and enhance electrical conductivity, resulting in improved supercapacitive electrochemical performance. The obtained CMDF electrode and the supercapacitor achieved remarkable maximum specific capacitance with superior long-term cycling stability. Overall, the CMDF derived from a problematic waste denim textile demonstrates the remarkable potential for utilization in the smart wearable field.

## Supplementary Information

Supplementary file1 (MP4 7432 kb)

Supplementary file2 (PDF 923 kb)
