# Supplementary material for: MXene Enhanced 3D Needled Waste Denim Felt for High-Performance Flexible Supercapacitors
Source: Nanomicro Lett. 2023 Nov 29;16:36. doi: 10.1007/s40820-023-01226-y (PMC10686952; doi:10.1007/s40820-023-01226-y)
Supplement: Supplementary file 2 — Supplementary file2 (PDF 923 kb) [file 40820_2023_1226_MOESM2_ESM.pdf]

Supporting Information for

MXene Enhanced 3D Needled Waste Denim Felt for High-Performance Flexible Supercapacitors

Wei Fan [1,*], Qi Wang [1], Kai Rong [1], Yang Shi [2], Wanxi Peng [3,*], Handong Li[4], Zhanhu Guo [4,*], Ben Bin Xu [4], Hua Hou[5], Hassan Algadi [6], Shengbo Ge [2,*]

[1] School of Textile Science and Engineering, Key Laboratory of Functional Textile Material and Product of the Ministry of Education, Xi'an Polytechnic University, Xi'an, Shaanxi 710048, P. R. China

[2] Jiangsu Co-Innovation Center of Efficient Processing and Utilization of Forest Resources, International Innovation Center for Forest Chemicals and Materials, College of Materials Science and Engineering, Nanjing Forestry University, Nanjing, Jiangsu 210037, P. R. China

[3] Henan Province International Collaboration Lab of Forest Resources Utilization, School of Forestry, Henan Agricultural University, Zhengzhou 450002, P. R. China

[4] Integrated Composites Lab, Department of Mechanical and Construction Engineering, Northumbria University, Newcastle Upon Tyne, NE1 8ST, UK

[5] College of Materials Science and Engineering, Taiyuan University of Science and Technology, Taiyuan 030024, P. R. China

[6] Department of Electrical Engineering, Faculty of Engineering, Najran University, Najran, 11001, Saudi Arabia

*Corresponding authors. E-mail: fanwei@xpu.edu.cn (Wei Fan); pengwanxi@163.com (Wanxi Peng); zhanhu.guo@northumbria.ac.uk (Zhanhu Guo); geshengbo@njfu.edu.cn (Shengbo Ge)

Supplementary Figures and Tables

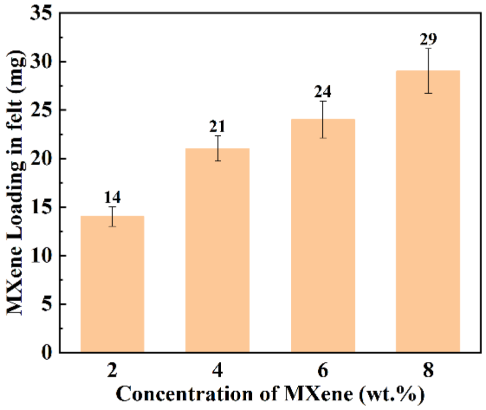

Fig. S1 Load amounts of MXene of DF after dipping different concentrations of MXene aqueous solution

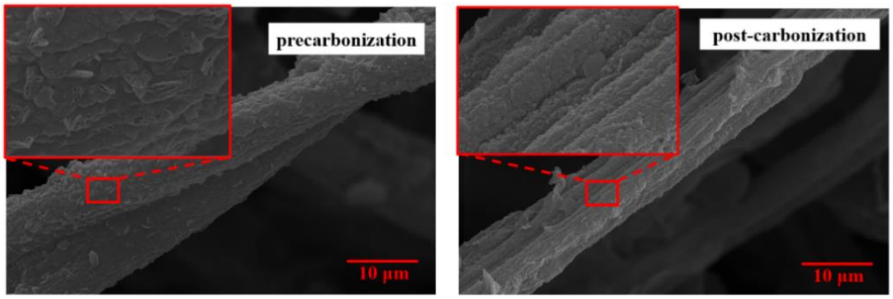

Fig. S2 MXene morphology on DF before and after carbonization

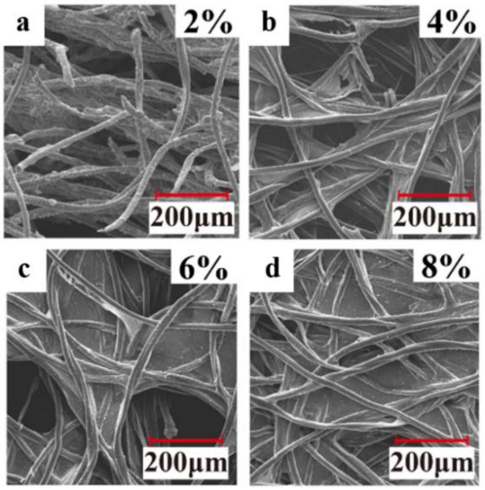

Fig. S3 SEM images of MDF impregnated from different MXene concentrations: (**a**) 2 wt.%, (**b**) 4 wt.%, (**c**) 6 wt.%, and (**d**) 8 wt.%, respectively

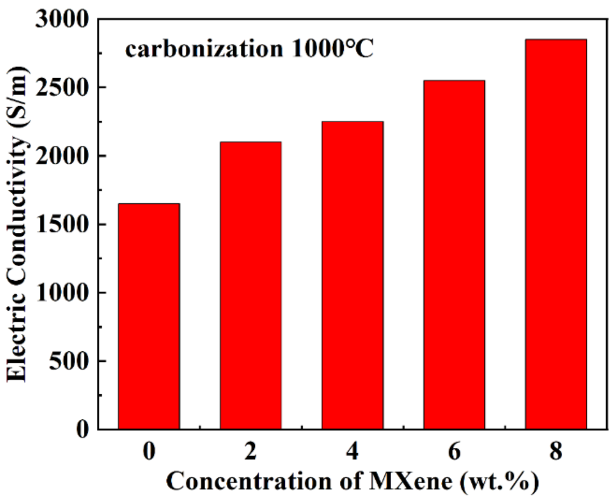

Fig. S4 Electrical conductivity of CMDF impregnated from different MXene concentrations

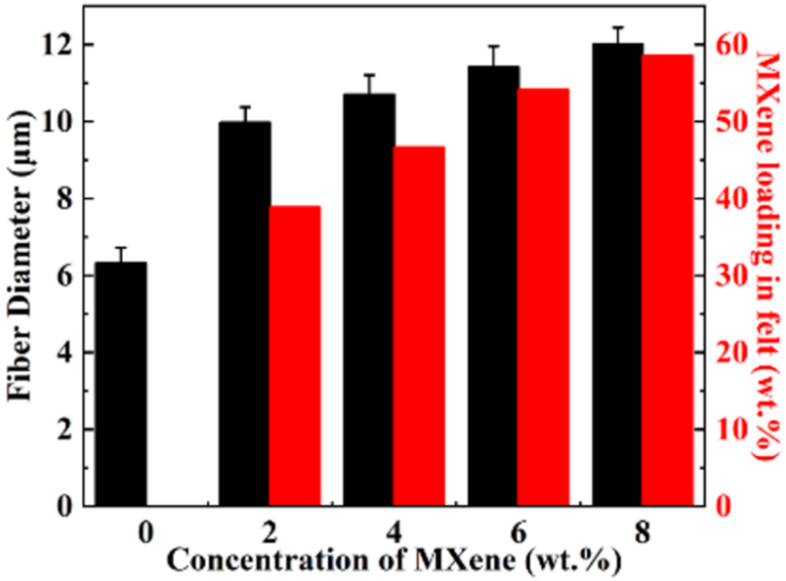

Fig. S5 Histogram of fiber diameter and MXene load in MDF impregnated from different MXene concentrations

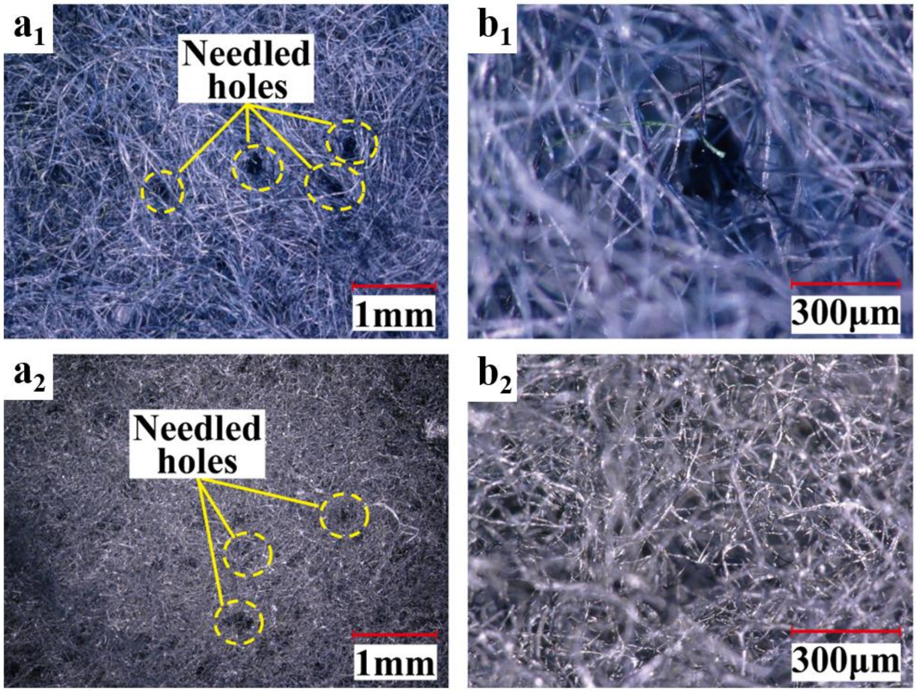

Fig. S6 SEM images of the DF at (**a1**) low resolution and (**b1**) high resolution; SEM images of the CDF at (**a2**) low resolution and (**b2**) high resolution

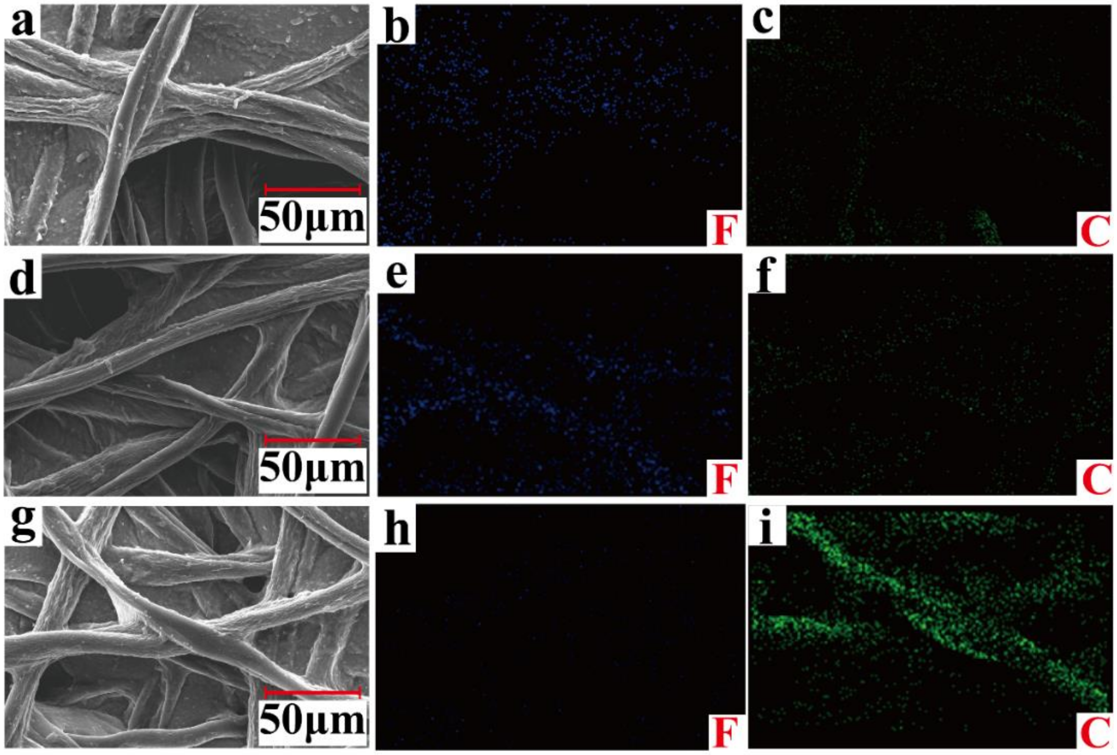

Fig. S7 EDX mapping results of CMDF at different carbonization temperatures: (**a- c**) 800 ℃, (**d-f**) 1000 ℃, (**g-i**) 1200 ℃

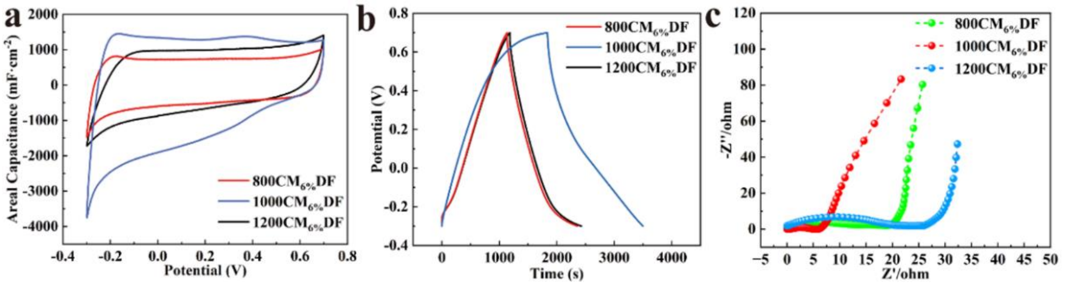

Fig. S8 (**a**) CV curves, (**b**) GCD curves and (**c**) EIS spectra of CMDF at different carbonization temperatures

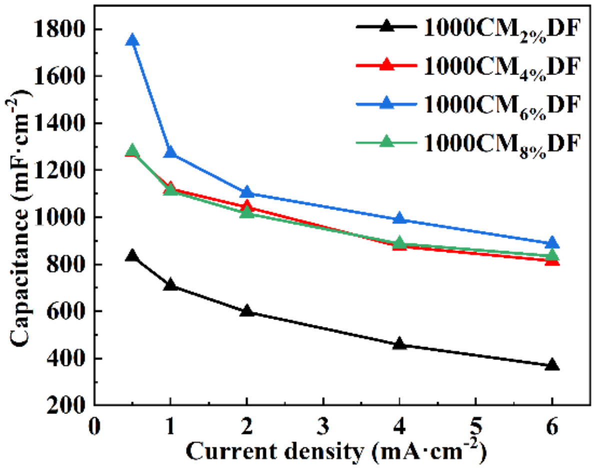

Fig. S9 The electrochemical performance of CMDF with different MXene loadings

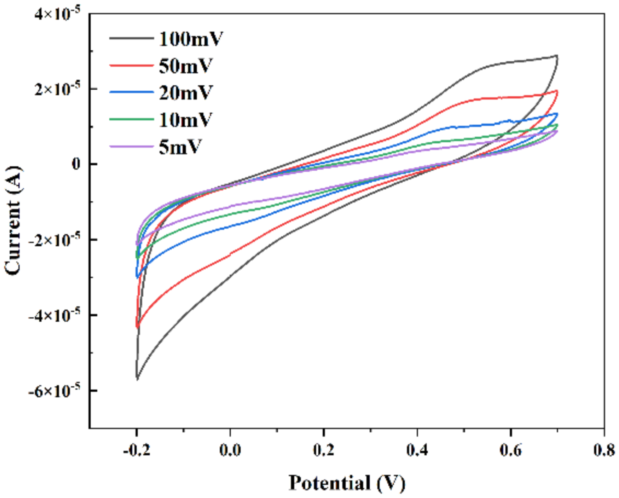

Fig. S10 CV plot of the supercapacitor assembled by CDF

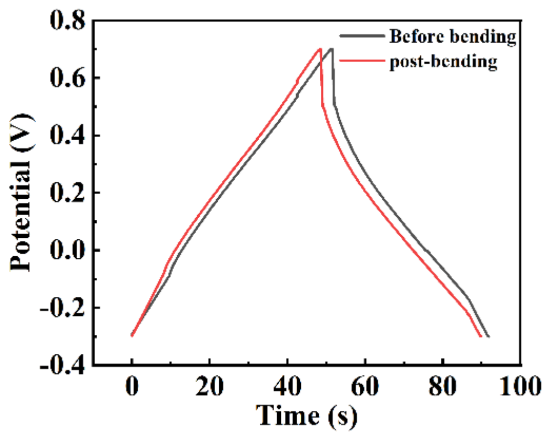

Fig. S11 GCD of the supercapacitor before and after 1000 cycles bending with 180 degress

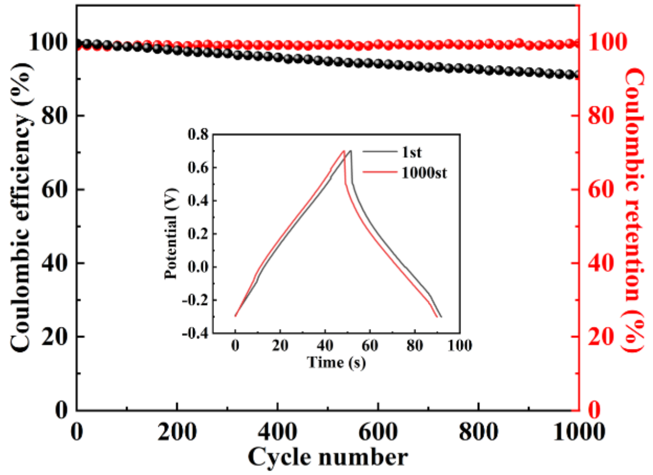

Fig. S12 GCD of the supercapacitor before and after folding for 1000 cycles

Table S1 Comparison of mass loading with other relevant works

Active materials	Substrate	Mass loading	Refs.
MXene	Silk-derived carbon cloth	2 mg cm^{-2}	[S1]
MXene	Silver-plated nylon fiber	0.8 mg cm^{-1}	[S2]
MXene	Cellulose Yarns	2.2 mg cm^{-1}	[S3]
MXene	Cotton yarn	0.62 mg cm^{-1}	[S4]
MXene	Cotton fabric	3 mg cm^{-2}	[S5]
MXene	denim waste fiber needle felts	30 mg cm^{-2}	This work
MXene	/	12 mF cm^{-2}	[1]
MXene	/	182 F g^{-1}	[2]
MXene	/	34.87 $mFcm^{-2}$	[3]

Supplementary References

[S1] M. Hu, T. Hu, R. Cheng et al., MXene-coated silk-derive d carbon cloth toward flexible electrode for supercapacitor application. J. Energy Chem. **27**(1), 161-166 (2018). https://doi.org/10.1016/j.jechem.2017.10.030

[S2] M. Hu, Z. Li, G. Li et al., All-solid-state flexible fiber-based mxene supercapacitors. Adv. Mater. Technol. **2**(10), 1700143 (2017). https://doi.org/10.1002/admt.201700143

[S3] S. Uzun, S. Seyedin, A.L. Stoltzfus et al., Knittable and washable multifunctional MXene-coated cellulose yarns. Adv. Funct. Mater. **29**(45), 1905015 (2019). https://doi.org/10.1002/adfm.201905015

[S4] K. Jost, D.P. Durkin, L.M. Haverhals et al., Natural fiber welded electrode yarns for knittable textile supercapacitors. Adv. Energy Mater. **5**(4), 1401286 (2015). https://doi.org/10.1002/aenm.201401286

[S5] Y. Li, Z. Lu, B.J. Xin et al., All-solid-state flexible supercapacitor of carbonized mxene/cotton fabric for wearable energy storage. Appl. Surface Sci. 528 (2020). https://doi.org/10.1016/j.apsusc.2020.146975

[1] C. Zhang, L. McKeon, M.P. Kremer, S.-H. Park, O. Ronan et al., Additive-free MXene inks and direct printing of micro-supercapacitors. Nat. Commun. **10**, 1795 (2019). https://doi.org/10.1038/s41467-019-09398-1

[2] J. Tang, W. Yi, X. Zhong, C. (John) Zhang, X. Xiao et al., Laser writing of the restacked titanium carbide MXene for high performance supercapacitors. Energy Storage Mater. **32**, 418–424 (2020). https://doi.org/10.1016/j. ensm.2020.07.028

[3] Y. Wu, D. Zhao, J. Zhang, A. Lin, Y. Wang et al., Microscale curling and alignment of $Ti_3C_2T_x$ MXene by confining aerosol droplets for planar micro-supercapacitors. ACS Omega **6,** 33067–33074 (2021). https:// doi.org/10.1021/acsomega.1c05373
